# N170 Amplitude to Rare Neutral Faces in an Oddball Condition Reflects Prediction Error

**DOI:** 10.1111/ejn.70264

**Published:** 2025-09-30

**Authors:** Xinyang Liu, Xueqiao Li, Piia Astikainen

**Affiliations:** ^1^ Department of Psychology University of Jyväskylä Jyväskylä Finland

**Keywords:** deviance detection, ERPs, facial expression, prediction error, visual mismatch negativity

## Abstract

Visual mismatch negativity (vMMN) of event‐related potentials (ERPs) reflects automatic change detection in serially presented stimuli under the predictive coding framework. Previous studies have shown modulations of P1 and N170 components in response to changes in facial expressions, though these studies have not fully controlled for stimulus probability and low‐level visual features. We recorded P1 and N170 to facial expressions and investigated their associations to vMMN in 36 participants of wide age range (19–65 years, M = 46.19, SD = ±13.07) and varying levels of depressive symptoms (Beck's Depression Index‐II, M = 15.06, SD = ±12.43). Neutral, happy, and sad faces were assigned as deviant (*p*_deviant_ = 0.14) and standard stimuli in separate oddball conditions allowing comparison of responses to physically identical stimuli in the deviant and standard positions. A control condition with seven basic facial expressions (*p* = 0.14 for each) and no repetitive stimuli served as a control for predictive processing. We found evidence of predictive deviance detection in face processing, indicated in the N170 amplitude, but only for neutral faces (deviant > standard and deviant > control). Depressive symptoms did not correlate with ERP amplitudes or latencies, while the N170 amplitude increased with age. These findings suggest that, under conditions controlling for low‐level features and stimulus probability, prediction error is robustly indexed by N170 amplitude in response to neutral faces. No such effects were observed for emotional (happy or sad) faces, or in earlier ERPs (P1) or latency measures.

## Graphical abstract

This study examined deviance detection of facial expressions using a flip‐flop oddball design with neutral and emotional (sad, happy) faces. N170, but not P1, amplitude to neutral deviants reflected prediction error and increased with age. The figure shows sad/neutral face conditions with waveforms and violin plots for neutral faces.

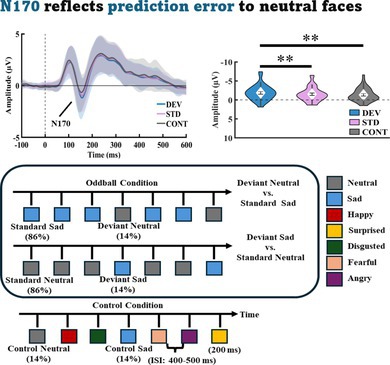

**Abbreviations**
ANCOVA: Analysis of covariance
ANOVA: Analysis of variance
BDI‐II: Beck Depression Inventory II
CONT: Control
ContHappy: Control happy
ContNeutral: Control neutral
ContSad: Control sad
DEV: Deviant
DevHappy: Happy deviant
DevNeutral: Deviant neutral
DevSad: Sad deviant
EEG: Electroencephalography
ERPs: Event‐related potentials
FDR: False discovery rate
ISI: Interstimulus interval
MMN: Mismatch negativity
STD: Standard
StHappy: Happy standard
StNeutral: Neutral standard
StSad: Sad standard
vMMN: Visual mismatch negativity

## 1 Introduction

Accurate and rapid detection of changes in facial expressions plays a crucial role in social cognition, facilitating sensitive human interactions. Facial expressions provide key cues about others' emotions and motivations, indicating for example whether an interaction is likely to be positive or negative. The ability to perceive changes in facial expressions, even without conscious attention, is essential for maintaining smooth and effective communication.

Event‐related potentials (ERPs), which represent time‐locked responses in electroencephalogram (EEG), are effective tools in revealing different processing stages of face perception. Visual ERP components P1 and N170, which primarily reflect low‐level feature encoding and the structural encoding of visual features, respectively, are the first major responses elicited in the cortical regions (Ganis et al. 2012). P1 is positive in polarity, maximum in amplitude in the occipital electrode sites, and peaks about 100 ms after stimulus onset (VanRullen and Thorpe 2001). N170 is a negative‐going deflection with maximum amplitude at lateral parietal electrode sites in response to face stimuli (Batty and Taylor 2003; Eimer 2000). N170 is a face‐sensitive component, as it is observed with a larger amplitude to faces than other objects (e.g., Blau et al. 2007; Earp and Everett 2013; Li 2021). While N170 amplitude is typically modulated by facial expression, this is less often the case for P1 (for reviews, see Pei et al. 2023; Schindler and Bublatzky 2020). For latency modulation, N170 latency has been shown to be modulated by facial expression, but not P1 latency (Batty and Taylor 2003).

ERP component called visual mismatch negativity (vMMN) can be used to investigate change detection in serially presented visual stimuli (for reviews, see Astikainen et al. 2022; Czigler 2014; Stefanics et al. 2015). vMMN, like its auditory counterpart (Näätänen et al. 1978), is elicited in the passive oddball condition where an infrequent (deviant) stimulus is interspersed with a frequently presented (standard) stimulus. Instead of reflecting the probability of the deviant stimulus alone, vMMN is suggested to reflect the detection of rare changes in a constant stimulus background, often referred to as deviance detection. vMMN has also been associated to prediction error (e.g., Astikainen et al. 2008; Czigler et al. 2019; File et al. 2017, 2020; Petro et al. 2020, 2023; Kimura et al. 2012; Kojouharova et al. 2019; Winkler and Czigler 2012) under the predictive coding theory (Friston 2010; Kimura 2012; Stefanics et al. 2014, 2015), which posits that the brain continuously makes predictions of the sensory environment based on past events and updates the predictive model when prediction errors are detected.

Previous studies have shown that rare emotional faces among neutral standard faces elicit ERP modulations, possibly confirming vMMN, at latency ranges that often correspond to P1 (e.g., Bruchmann et al. 2022; Li et al. 2023) and N170 (e.g., Astikainen and Hietanen 2009; Stefanics et al. 2012; Wu et al. 2017; Zhang et al. 2021) although sometimes later latencies have shown effects, too (e.g., Schindler et al. 2020, for emotional changes see, Schlüter and Bermeitinger 2017). However, most of the previous studies have compared responses to physically different deviant and standard faces (Astikainen and Hietanen 2009; Astikainen et al. 2013; Kovarski et al. 2017, 2022; Kuehne et al. 2019; Li et al. 2012; Liu et al. 2015, 2016; Soshi et al. 2015; Susac et al. 2004, 2010; Zhao and Li 2006; see, however, Gayle et al. 2012; Kaffes et al. 2023; Kreegipuu et al. 2013; Vogel et al. 2015; Wang et al. 2016; Zeng et al. 2022). Therefore, the responses could reflect different physical features of the stimuli instead of deviance detection in facial expressions. To contribute to this gap in the literature, here we will include stimulus conditions where a neutral face serves as deviant stimulus among emotional standard faces, and vice versa. This will allow us to compare responses to each facial expression as standard and deviant stimulus (here the expressions will be neutral, happy, and sad). Importantly, we will use an equiprobable control condition (first reported in context of the auditory MMN, Schröger and Wolff 1996) to investigate whether the possible differential responses at P1 and N170 latencies in the oddball condition reflects prediction error. In this control condition different facial expressions (neutral face and the six basic facial emotions) occur with equal probability and are presented without repetitions or predictability. Since the probability for each facial expression is the same as that for the deviant stimulus in the oddball condition, larger responses to the oddball deviant stimulus than to control stimulus of the same emotion reflects prediction error signal. Only a few previous studies have used equal probability control condition in the vMMN studies of facial expressions, but they have either compared physically different stimuli (Kimura et al. 2009; Kovarski et al. 2017, 2021, 2022; Li et al. 2012) or their control and deviant stimuli have had different probabilities in their series (Astikainen et al. 2013). Therefore, our study is important in controlling for the effects of physical features of the stimuli while contrasting responses to stimuli having the same probability but different contexts: predictable (repetitive standard faces in the oddball condition) and unpredictable (ever‐changing facial emotions in the equiprobable condition).

We also aim to investigate the effects of depressive symptoms on ERPs to faces. Negative bias toward sad contents in depression is a commonly reported phenomenon in behavioral (e.g., Peckham et al. 2010; Suslow et al. 2020) and brain studies (e.g., Bistricky et al. 2014; Dai and Feng 2012; Zhang et al. 2016; Zhao et al. 2015). Previous ERP studies using visual oddball task have also shown that depressive disorder and depressive symptoms in preclinical participant groups are associated with negative bias to sad faces. For example, larger P1 responses were found to sad faces than to happy or neutral faces in the depressed group (Ruohonen, Alhainen, and Astikainen 2020; Xu et al. 2018; for absent negative bias in P1, see Dai et al. 2016; Zhao et al. 2015). Likewise, larger N170 responses to sad faces than to happy and/or neutral faces in depressed group have been reported (Chen et al. 2014; Wu et al. 2016; Zhang et al. 2016; Zhao et al. 2015; for absent negative bias in N170, see Chang et al. 2010; Ruohonen, Alhainen, and Astikainen 2020). Negative bias is not only observed as increased responses to negative contents, but also as decreased responses to positive contents compared with responses to neutral ones (e.g., smaller responses to happy faces than to neutral faces, Zhang et al. 2016). We therefore selected sad and happy faces as emotional expressions for our study. We will also investigate possible effect of age on responses because previous studies have indicated that depression effects can be partly parallel with the aging effects (for visual ERPs to faces, see Csizmadia et al. 2021; for auditory oddball condition, see Kangas et al. 2024; Ruohonen, Kattainen, et al. 2020).

We expect that N170, but not necessarily P1 component, reflects deviance detection in facial expressions confirming prediction error signal. This would be evidenced by a larger response amplitude for deviant than standard stimuli in oddball condition and also a larger response amplitude for deviant than control stimuli. We hypothesize that in addition to stimulus type (deviant, standard, control) also facial expression will affect the responses, especially N170 amplitude and latency (e.g., Hinojosa et al. 2015; Batty and Taylor 2003), and it is therefore not known whether prediction error signals are elicited similarly to neutral, sad, and happy faces. Due to the limited number of studies exploring P1 or N170 latency changes in the context of deviance detection (Liu et al. 2016; Zhao and Li 2006), we did not specify a priori hypotheses for latency effects. Nonetheless, theoretical models of predictive coding suggest that deviant stimuli—by violating established expectations—should invoke additional hierarchical processing and model updating, which could plausibly delay neural responses compared to standards (Friston 2010).

In the analysis, comparisons are made between physically identical stimuli by comparing the response amplitudes for the same facial emotion (neutral, sad, and happy) as deviant, standard, and control stimulus. In addition, for the sake of comparability to previous studies, we report in supplementary materials analyses based on comparisons between standard and deviant stimulus responses obtained within each oddball condition (comparing thus responses to physically different stimuli). If the physical features of the face stimuli affect the results, the response patterns can be different from the main analysis.

## 2 Method

### 2.1 Participants

The sample size of this study was estimated to investigate the main effect of stimulus type for each facial expression, but not for correlation analysis. We calculated the sample size by conducting a priori analysis in G*Power (Faul et al. 2007, version 3.1.9.7). For P1 and N170 components, a repeated‐measures ANOVA was selected to test the main effect of stimulus type with three levels was selected as the statistical test. The statistical power (1 − β) = 0.80, the significance level of alpha = 0.05 and the nonsphericity correction = 1. The effect size was set on the specification as in SPSS (IBM SPSS Statistics; IBM Corporation, NY, USA). A medium effect size (*η*_p_^2^ = 0.060; Cohen 1988) was referred to for the estimation of the sample size in the present study. Based on the calculation, 27 participants would be enough to obtain a medium effect size for the main effect of stimulus type.

The participants of the present study participated in a larger study investigating efficacy of a brief psychological intervention for depressive disorder (Kyllönen et al. 2018). We have previously reported differences in brain responses to facial expressions between groups of depressed participants and never‐depressed controls as well as their association to the treatment response in depression group (Ruohonen, Alhainen, and Astikainen 2020). Here, we report data from the same sample, which is partly reported in Ruohonen, Alhainen, and Astikainen (2020). While Ruohonen, Alhainen, and Astikainen (2020) utilized the data from the stimulus conditions having emotional (sad and happy) deviant stimuli in oddball conditions, here, we use these together with two other oddball conditions with neutral deviant stimuli among emotional (sad and happy) standard stimuli, and an equal probability control condition, which have not been reported before. All five stimulus conditions were available from 48 of all participants (*n* = 52). Since here the focus is on predictive processing, we combine the data from depressed and control group and examine depression scores as a covariate in follow‐up analyses.

The recruitment was conducted separately for the depressed and control group and realized via email lists at the University of Jyväskylä and with an advertisement in the local newspaper. The inclusion criteria for all the depressed and nondepressed participants were age of 18–65 years, right‐handedness, normal hearing, and normal or corrected‐to‐normal vision. Exclusion criteria were self‐reported: 1) current substance abuse or addiction to drugs and intoxicants, including alcohol, and 2) current or previous diagnosis of psychiatric disorder other than possible depressive disorder, neurological disorder, or neurological injury. All the participants were asked to fill in the Beck's Depression Inventory II (BDI‐II; Beck et al. 1996) to evaluate their current depression symptoms.

The participants volunteering for the depression group were screened for clinical depression based on the International Classification of Diseases and Related Health Problems, 10th Revision (ICD‐10; World health organization, 2010) by a physician independent of the study. Exclusion criteria were as follows: 1) serious suicide risk (because a wait‐list control group for intervention was applied); 2) depression with psychotic features; 3) current substance abuse or addiction to drugs and intoxicants, including alcohol; and 4) diagnosis of psychotic disorder, bipolar disorder, eating disorder, or history of neurological injury or disease.

Thirty‐seven clinically depressed individuals and 31 never‐depressed controls volunteered for the original study. In the current study, of the 48 participants (11 male, M = 48.08 years, SD = ±13.43) having all the required datasets, 30 were from the depressed group, and 18 participants were from the nondepressed control group. Among the 48 participants of the present study, 12 participants were excluded due to excessive eye blinks or other artefacts in the EEG data (in one or more stimulus conditions). Please note that the percentage of participants with rejected data was larger here than in Ruohonen, Alhainen, and Astikainen (2020) because we utilized five versus two stimulus conditions, respectively, in these studies. Finally, 36 participants' data (7 male, M = 46.19 years, SD = ±13.07) were included in the data analysis, and the mean BDI‐II score for all participants was 15.06 (SD = ± 12.43, range 0–42). Among the 36 participants, 12 were from the control group, and 24 were from the depressed group. All the participants signed a written informed consent form before participating. The research protocol was approved by the ethical committee of the University of Jyväskylä.

### 2.2 Stimuli

Twenty‐eight grayscale pictures of emotional faces were selected from Pictures of Facial Affect (Ekman and Friesen 1976), in which four actors (two females and two males) demonstrated seven facial expressions (disgusted, surprised, sad, angry, fearful, happy, and neutral). All seven expressions were included in the equal probability control condition, while neutral, sad, and happy faces were used in the oddball conditions. The size of the face image was 728 × 1080 pixels, occupying an area of 11 × 16° of visual angle. The experiment was controlled using E‐Prime version 2.0.8.90 (Psychology Software Tools Inc., Sharsburg, MD, USA), a Dell 5500 computer and a 23‐in. monitor (Asus VG236 series H; refresh rate = 120 Hz; display resolution = 1920 × 1080) were used to present the stimuli.

**FIGURE 1:**
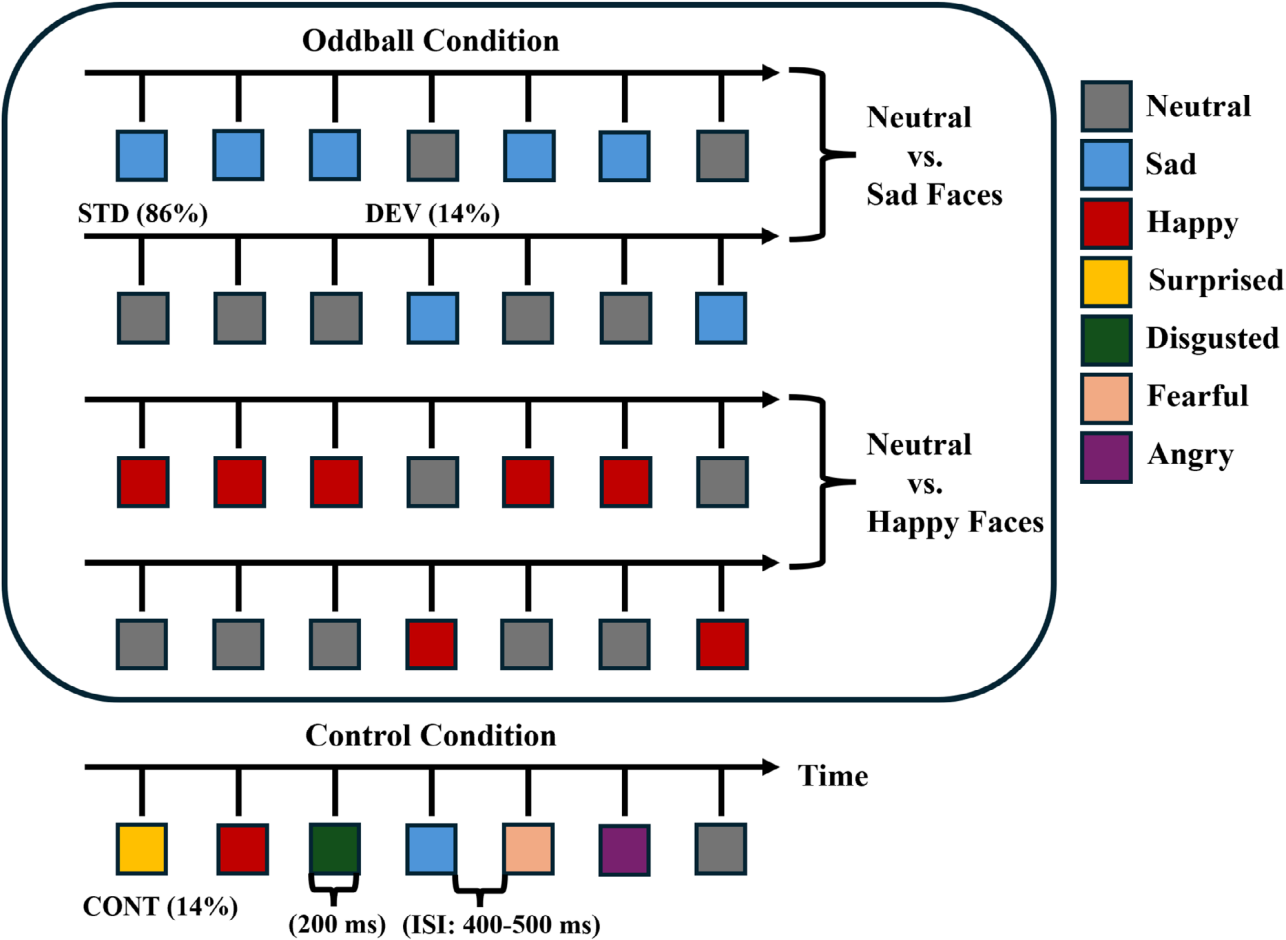
Schematic description of the stimulus presentation in the oddball and control conditions. CONT = control, DEV = deviant, ISI = interstimulus interval (offset‐to‐onset), STD = standard. Stimulus duration was 200 ms in all the conditions.

The stimulus conditions are depicted in Figure 1. In the oddball condition, the deviant stimulus was infrequently interspersed between frequently presented standard faces. Both neutral and emotional faces were used as deviant and standard stimuli. When the deviant stimulus was an emotional face (either happy or sad), the standard stimulus was a neutral face. Conversely, when the deviant stimulus was a neutral face, the standard stimulus was an emotional face (either happy or sad). In the oddball conditions, the stimuli were presented in a pseudo‐random order, with at least two standard stimuli between consecutive deviant stimuli. Out of 560 stimuli, there were 80 deviants (*p* = 0.14). In the control condition, all seven expressions (80 trials for each expression) were presented pseudo‐randomly, with no immediate repetitions of stimuli from the same emotion category and with an equal probability for each facial expression (*p* = 0.14). For statistical analysis, only the data from sad, happy, and neutral faces in the control condition was used as these three emotions were applied in the oddball conditions. The number of trials for each emotion category in the control condition was matched to the number of trials for the deviants in each oddball condition. The facial identity in the pictures varied from trial to trial in both the oddball and control conditions. Each stimulus was presented for 200 ms with an interstimulus interval (ISI, offset‐to‐onset) ranging between 400–500 ms. A randomly jittered ISI—consistent with our previous study (Ruohonen, Alhainen, and Astikainen 2020)—was employed to reduce temporal predictability and minimize anticipatory neural responses in early ERP components such as P1 and N170.

### 2.3 Procedure

During the experiment, the participants sat in a comfortable chair in a dimly lit, soundproof and electrically shielded room and were monitored via a video camera. Facial images were presented on a screen, which was approximately 1 meter away from the participant. At the same time, an audiobook was playing from the ceiling loudspeaker above them. The participants were asked to fixate their gaze on the middle of the screen, focus on the audiobook, and not to pay attention to the face stimuli. Questions about the story were asked between the blocks to ensure that participants did focus on the audiobook.

The five stimulus blocks (four different oddball blocks and a control block) were presented in a counterbalanced order between the participants (Figure 1). In oddball conditions, there were 80 deviant faces and 480 standard faces in each stimulus block. The equal probability condition included presentation of 560 faces, 80 for each facial expression.

### 2.4 EEG Recording

EEG data were measured by the Net Amps 200 (Electrical Geodesic Inc., Eugene, OR, USA) amplifier with a 128‐channel HydroCel Geodesic Sensor Net (HydroCel Geodesic Sensor Net, Electric Geodesic Inc., USA). The data were recorded with Net Station software (version 4.2.1). The sampling rate was 1000 Hz, and the recorded data were filtered online from 0.1 to 400 Hz. During the recording, impedances were kept below 20 kΩ, and the vertex electrode (Cz) was used as a reference electrode.

### 2.5 EEG Preprocessing

The preprocessing of the EEG data was carried out with Analyzer (BrainVision Analyzer, Version 2.2.2, Brain Products GmbH, Gilching, Germany). Channels with excessive noise were interpolated via the spherical spline method (Perrin et al. 1989). Following interpolation, a 0.1–30‐Hz band pass filter was applied to the EEG signal. The data were then segmented into epochs from 100 ms before and 600 ms after stimulus onset. Baseline correction was performed on all the epochs by subtracting the mean voltage of the 100‐ms prestimulus period from each data point within the epoch. Bad epochs, including eyeblinks and other artefacts with different sources (i.e., muscle activity), were automatically detected and removed when they exceeded ±150 μV. Finally, the EEG data were re‐referenced offline to an average of all channels.

For each stimulus type (deviant, standard, control), epochs were averaged separately for brain response to the facial expressions (sad deviant, happy deviant, and neutral deviant; sad standard, happy standard, neutral standard; control sad, control happy, control neutral). Specifically, standard stimuli immediately preceding the deviant stimuli were selected for data analysis, leading to the same number of trials for standard and deviant stimuli. As the neutral faces were presented in all four oddball blocks, and sad and happy faces both only in two blocks, we selected the odd trials for neutral deviant and standard faces from the blocks they were applied. To achieve high quality data, a minimum of 40 epochs was required for each facial expression and for all stimulus types, or otherwise the data for that participant were excluded from the analysis. In the current study, amplitudes of P1 and N170 components were investigated. Time windows and electrode clusters over the left and right occipital/parieto‐occipital sites were selected for each component based on previous literature (Astikainen et al. 2013; Li et al. 2023) and visual inspection of the grand averaged waveforms and topographies. For P1, channels 58, 59, 65, 66, 70, 83, 84, 90, 91, and 96 and for N170, channels 50, 57, 58, 64, 95, 96, 100, and 101 were chosen. For the latency analysis, the time point of the maximum peak amplitude for each component was detected for all the electrodes in the cluster and for each participant: for P1, the maximum amplitude of the positive‐going peak within 80–120‐ms latency, and for N170, the maximum amplitude of the negative going peak within 130–210‐ms latency. For the amplitude analysis, the mean amplitude value was calculated within a 20‐ms time window (± 10 ms) centered on the peak separately for P1 and P170 component. The peak latencies and amplitudes were calculated as mean value within the electrodes in each cluster (P1 and N170).

### 2.6 Statistical Analysis

The statistical analysis was conducted via IBM SPSS Statistic Version 26 (Armonk, NY: IBM Corporated). Separately for the P1 and N170 amplitude and latency, a one‐way repeated measures analysis of variance (ANOVA) with within‐subject variable stimulus type (deviant, standard, control) was applied for each facial expression (sad, happy, neutral) to investigate the predictive processing of different facial expressions. Bonferroni correction (Bonferroni 1936) was used to correct the *p* values whenever more than two ANOVAs were conducted. Post hoc analysis via two‐tailed paired samples *t* tests were conducted separately for each expression if a significant main effect was found. For main effect, *p* values smaller than 0.050 were considered significant. False discovery rate (FDR; Benjamini and Yekutieli 2001) procedure was used to correct the *p* values (*p*_FDR‐corrected_) whenever more than two *t* tests or correlations were conducted.

JASP (version 0.19.3, JASP Team 2025) was used to compute Bayes factors to assess support for the alternative versus the null hypothesis in post hoc *t* tests (Rouder et al. 2009). The Bayes factor represents the odds ratio between the alternative and null hypotheses: Values greater than 1 indicate support for the alternative hypothesis, whereas values less than 1 favor the null hypothesis. Default Cauchy priors (scale = 0.707) for effect sizes were used, as implemented in JASP.

Since modulational effects by depressiveness and aging on ERP responses have been reported in previous studies (for depressiveness effects, see e.g., Ruohonen, Alhainen, and Astikainen 2020; Xu et al. 2018; Chen et al. 2014; Wu et al. 2016; Zhang et al. 2016; Zhao et al. 2015; for aging effects, see Csizmadia et al. 2021; Ruohonen, Kattainen, et al. 2020), we conducted repeated measures analysis of covariance (ANCOVA) with depressiveness (BDI‐II scores) and age as separate covariates, to investigate their influence on the original ANOVA results. If a significant main effect found in the original ANOVA was eliminated after including a covariate, or if no main effect was originally present but emerged when the covariate was added, this would suggest that the covariate modulated the original effects. In such cases, the adjusted *p* values are reported, and a correlation analysis using Pearson's correlation coefficient (*r*) was performed to further explore the direction of these effects. To be able to compare the results with the previous studies, which have compared responses to different facial expressions presented as standard and deviant (typically emotional deviants vs. neutral standards), a separate analysis of the traditional vMMN‐like difference waves between the deviant and standard stimuli within each oddball block was also conducted and reported here as supplementary materials. One‐sample *t* tests were applied to the differential responses (deviant minus standard) for each component and expression against zero.

Partial eta squared (*η*_p_^2^) is reported as the effect size estimates for ANOVAs and ANCOVAs, while Cohen's *d* (Cohen 1988) with pooled standard deviation is used as the effect size estimates for *t* tests.

## 3 Results

Amplitude and latency values for P1 and N170 are presented separately for each facial expression and stimulus type in Tables 1 and 2, respectively.

**TABLE 1:** Mean and standard deviation of P1 amplitude (μV) and latency (ms), presented separately for each facial expression and stimulus type, as well as averaged across all expressions and stimulus types.

	Expression	Deviant	Standard	Control	Average across stimulus types
Amplitude	Sad	4.38 (2.39)	4.28 (2.26)	4.32 (2.33)	4.33 (2.27)
	Happy	4.12 (2.45)	4.13 (2.50)	4.29 (2.16)	4.18 (2.31)
	Neutral	4.24 (2.47)	4.16 (2.38)	4.38 (2.20)	4.26 (2.29)
	Average across expressions	4.24 (2.41)	4.19 (2.32)	4.33 (2.19)	
Latency	Sad	104.04 (8.72)	109.26 (14.20)	100.98 (9.71)	104.76 (9.90)
	Happy	103.82 (9.13)	103.66 (9.62)	100.53 (9.02)	102.67 (8.95)
	Neutral	102.15 (8.58)	102.24 (8.63)	100.35 (9.04)	101.58 (8.31)
	Average across expressions	103.33 (8.49)	105.05 (9.69)	100.62 (8.97)	

**TABLE 2:** Mean and standard deviation of N170 amplitude (μV) and latency (ms), presented separately for each facial expression and stimulus type, as well as averaged across all expressions and stimulus types.

	Expression	Deviant	Standard	Control	Average across stimulus types
Amplitude	Sad	−1.62 (1.95)	−1.72 (1.91)	−1.50 (1.67)	−1.61 (1.79)
	Happy	−2.00 (2.00)	−1.74 (2.08)	−1.51 (2.06)	−1.75 (1.99)
	Neutral	−1.85 (1.97)	−1.50 (1.86)	−1.33 (1.76)	−1.56 (1.82)
	Average across expressions	−1.82 (1.92)	−1.65 (1.91)	−1.45 (1.79)	
Latency	Sad	160.40 (13.13)	159.91 (12.38)	158.05 (14.50)	159.45 (12.88)
	Happy	159.60 (11.90)	159.39 (12.53)	158.43 (14.81)	159.14 (12.45)
	Neutral	160.32 (12.02)	158.94 (12.04)	156.19 (13.96)	158.48 (12.23)
	Average across expressions	160.10 (11.60)	159.41 (11.77)	157.56 (14.16)	

### 3.1 P1 Component

**FIGURE 2:**
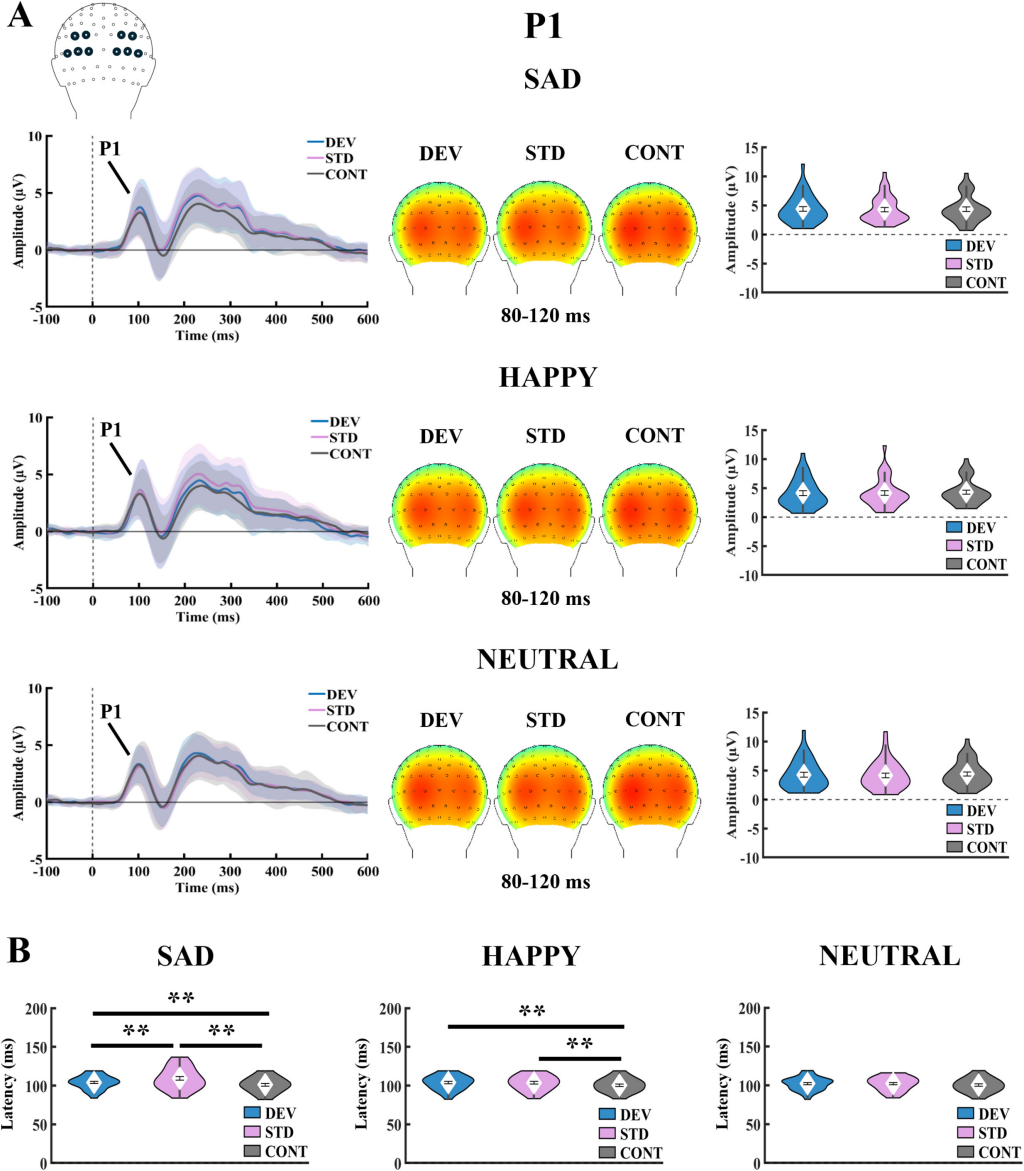
A) Stimulus type effect for P1 amplitude under each facial expression condition. Grand averaged ERP waveforms, topographies, and violin plots. The violin plots represent the mean, SEM, and the distribution (density) of the data. ERP waveforms represent mean values and SEM (shaded area) averaged across the marked electrodes. The blue line represents deviant stimulus, the purple line represents standard stimulus, and the grey line represents the control stimulus. The topographies were extracted from the 80–120‐ms poststimulus time window used to determine individual peak amplitudes for the P1 component. No significant effect for stimulus type was found for P1 amplitude. B) Stimulus type effect for P1 latency under each facial expression condition. The violin plots represent the mean, SEM, and the distribution (density) of the data. CONT = control stimulus, DEV = deviant stimulus, SEM = standard error of the mean, STD = standard stimulus. **p < 0.01.

For P1 amplitude (Figure 2A), a one‐way repeated measures ANOVA found no main effect of stimulus type on responses to sad, *F*(2, 70) = 0.23, *p* = 0.796, *η*_p_^2^ = 0.01, happy, *F*(2, 70) = 0.75, *p* = 0.477, *η*_p_^2^ = 0.02, or neutral faces, *F*(2, 70) = 0.94, *p* = 0.380, *η*_p_^2^ = 0.03.

For P1 latency (Figure 2B), a significant effect of stimulus type was found for sad, *F*(2, 70) = 16.20, *p*_Bonf‐corrected_ < 0.003, *η*_p_^2^ = 0.32, and happy faces, *F*(2, 70) = 14.79, *p*_Bonf‐corrected_ < 0.003, *η*_p_^2^ = 0.30, but not for neutral faces, *F*(2, 70) = 3.67, *p*_Bonf‐corrected_ = 0.09, *η*_p_^2^ = 0.10. BDI‐II scores as a covariate removed the main effect of stimulus type for happy face (*p* = 0.765). However, no correlation was found between BDI‐II scores and any stimulus type in the happy condition (all *p* values > 0.101).

Paired‐samples *t* tests were conducted as post hoc analyses to compare responses to different stimulus types separately for sad and happy conditions (Table 3). A significant latency difference was found between sad deviant and sad standard faces, with deviant stimuli eliciting shorter latencies (*p*_FDR‐corrected_ = 0.005, BF_10_ = 8.38). No difference was found between latencies for happy deviant and standard faces (*p*_FDR‐corrected_ = 0.809, BF_10_ = 0.18). Response latencies were shorter for control than deviant (sad: *p*_FDR‐corrected_ = 0.002, BF_10_ = 363.67; happy: *p*_FDR‐corrected_ = 0.002, BF_10_ = 376.31) and standard stimuli (sad: *p*_FDR‐corrected_ = 0.002, BF_10_ = 614.40; happy: *p*_FDR‐corrected_ = 0.002, BF_10_ = 658.78).

**TABLE 3:** P1 latency results. Results of the post hoc (paired‐samples) *t* tests investigating the stimulus type effect for sad and happy face responses. The direction of each significant comparison (e.g., DEV<STD) is indicated. For P1, a shift toward positive polarity indicates a larger amplitude.

	Comparison	*t*	*p*_FDR‐corrected_	95% CI	Cohen's *d*
Sad	DEV < STD	3.04	**0.005**	[−8.71, −1.73]	0.44
	DEV > CONT	4.53	**0.002**	[1.69, 4.43]	0.33
	STD > CONT	4.72	**0.002**	[4.72, 11.83]	0.68
Happy	DEV vs. STD	0.24	0.809	[−1.18, 1.50]	0.02
	DEV > CONT	4.54	**0.002**	[1.82, 4.76]	0.36
	STD > CONT	4.75	**0.002**	[1.79, 4.46]	0.34

*Note:* Degrees of freedom = 35; *t* values, *p* values (FDR‐corrected), and Cohen's *d* for effect size. Significant *p* values are marked in bold.

Abbreviations: CONT = control stimulus, DEV = deviant stimulus, STD = standard stimulus.

### 3.2 N170 Component

**FIGURE 3:**
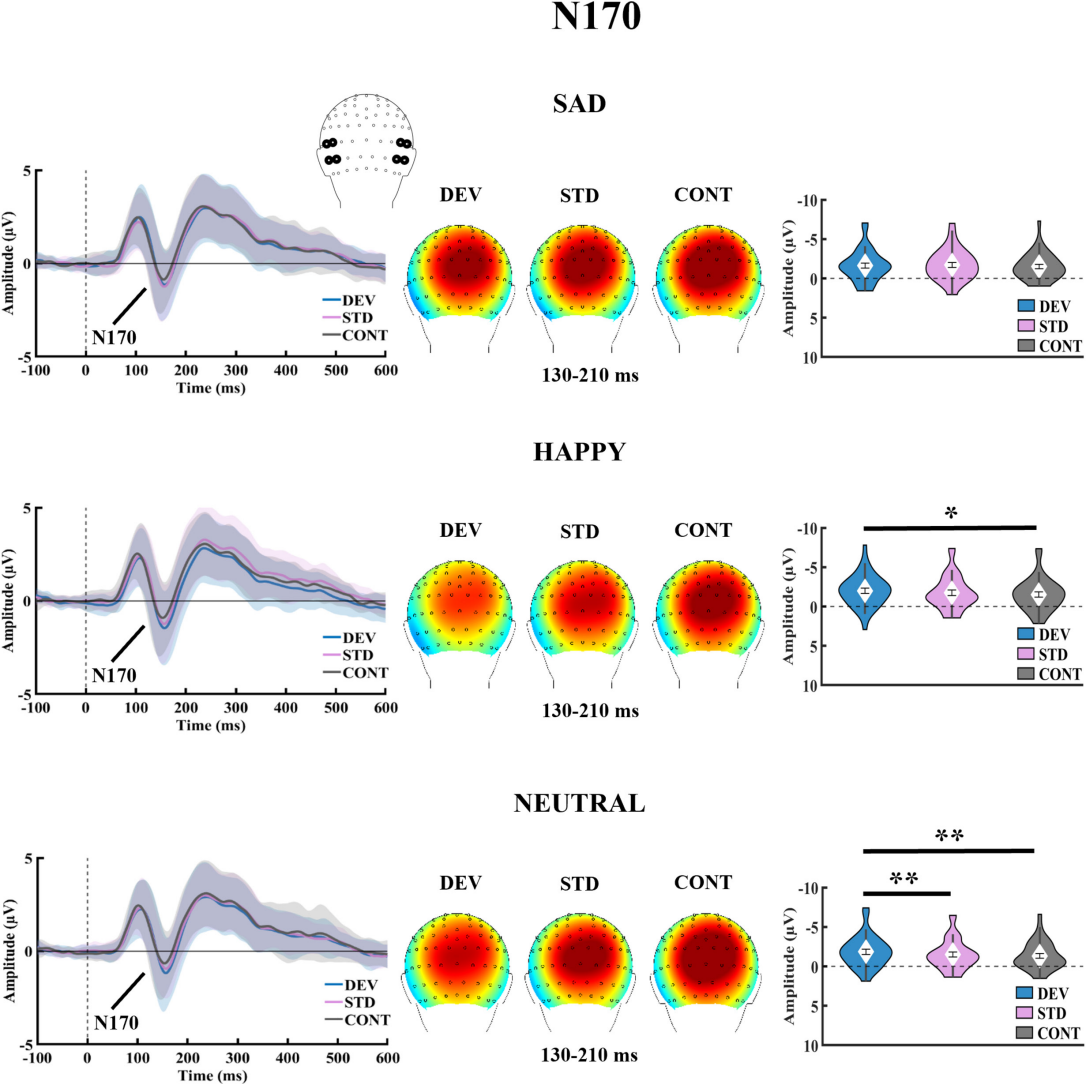
Stimulus type effect for N170 amplitude under each facial expression condition. Grand averaged ERP waveforms, topographies, and violin plots. The violin plots represent the mean, SEM, and the distribution (density) of the data. ERP waveforms represent mean values and SEM (shaded area) averaged across the marked electrodes. The blue line represents deviant stimulus, the purple line represents standard stimulus, and the grey line represents the control stimulus. The topographies were extracted from the 130–210‐ms poststimulus time window used to determine individual peak amplitudes for the N170 component. CONT = control stimulus, DEV = deviant stimulus, SEM = standard error of the mean, STD = standard stimulus. *p < 0.05, **p < 0.01.

For amplitude, one‐way repeated measures ANOVAs investigating the effect of stimulus type separately for each facial expression (Figure 3) indicated a main effect for happy, *F*(2, 70) = 5.96, *p*_Bonf‐corrected_ = 0.012, *η*_p_^2^ = 0.15, and neutral, *F*(2, 70) = 9.39, *p*_Bonf‐corrected_ < 0.003, *η*_p_^2^ = 0.21, but not for sad faces, *F*(2, 70) = 1.41, *p* = 0.250, *η*_p_^2^ = 0.04. Larger N170 amplitude for deviant stimuli compared with standard stimuli was found only for neutral faces (*p*_FDR‐corrected_ = 0.005, BF_10_ = 11.92), but not happy faces (*p*_FDR‐corrected_ = 0.083, BF_10_ = 1.04). Deviant faces elicited larger N170 amplitude than control faces both for happy (*p*_FDR‐corrected_ = 0.012, BF_10_ = 8.60) and neutral faces (*p*_FDR‐corrected_ = 0.003, BF_10_ = 39.07). Results of paired‐sample *t* tests comparing the response amplitudes of different stimulus types separately for happy and neutral faces are presented in Table 4.

**TABLE 4:** N170 amplitude results. Results of the post hoc (paired‐samples) *t* tests investigating stimulus type effect for happy and neutral face responses. The direction of each significant comparison (e.g., DEV > STD) is indicated. For N170, a shift toward negative polarity indicates a larger amplitude.

	Comparison	*t*	*p*_FDR‐corrected_	95% CI	Cohen's *d*
Happy	DEV vs. STD	1.99	0.083	[−0.52, 0.01]	0.13
	DEV > CONT	3.05	**0.012**	[−0.80, −0.16]	0.24
	STD vs. CONT	1.74	0.091	[−0.49, 0.04]	0.11
Neutral	DEV > STD	3.20	**0.005**	[−0.57, −0.13]	0.18
	DEV > CONT	3.68	**0.003**	[−0.82, −0.24]	0.28
	STD vs. CONT	1.52	0.139	[−0.41, 0.06]	0.09

*Note:* Degrees of freedom = 35 *t* values, *p* values (FDR‐corrected), and Cohen's *d* for effect size are reported. Significant *p* values are marked in bold.

Abbreviations: CONT = control stimulus, DEV = deviant stimulus, STD = standard stimulus.

BDI‐II scores as a covariate removed the main effect of stimulus type for N170 amplitude for happy faces (*p* = 0.114). However, no correlation was found between BDI‐II scores and any stimulus type in the happy face condition (all *p* values > 0.790).

**FIGURE 4:**
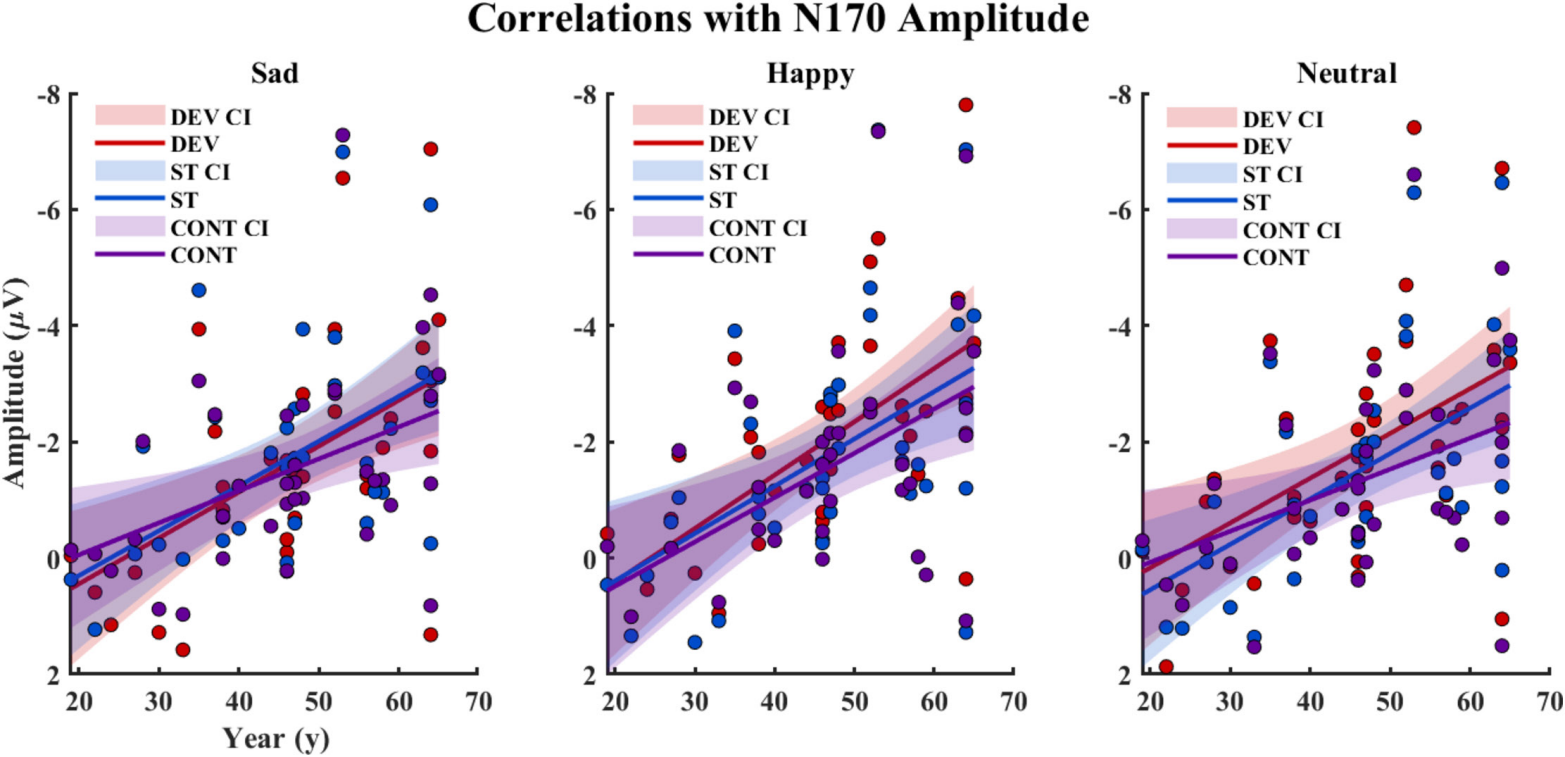
Correlation between age and N170 amplitude separately for different facial expressions. CI = 95% confidence interval, CONT = control stimulus, DEV = deviant stimulus, STD = standard stimulus.

In addition, age as a covariate removed the main effect of stimulus type for happy (*p* = 0.882) and neutral (*p* = 0.090) face responses. Correlation analysis found significant correlations between age and all stimulus types and expressions: deviant sad (*r* = −0.53, *p*_FDR‐corrected_ = 0.002), standard sad (*r* = −0.53, *p*_FDR‐corrected_ = 0.002), control sad (*r* = −0.43, *p*_FDR‐corrected_ = 0.010); deviant happy (*r* = −0.60, *p*_FDR‐corrected_ = 0.001), standard happy (*r* = −0.51, *p*_FDR‐corrected_ = 0.002), control happy (*r* = −0.49, *p*_FDR‐corrected_ = 0.004); deviant neutral (*r* = −0.51, *p*_FDR‐corrected_ = 0.002), standard neutral (*r* = −0.55, *p*_FDR‐corrected_ = 0.002), control neutral (*r* = −0.40, *p*_FDR‐corrected_ = 0.017). Correlations between N170 amplitude and different stimulus types for each expression are depicted in Figure 4.

For N170 latency, no main effect of stimulus type was found for sad, *F*(2, 70) = 2.89, *p* = 0.062, *η*_p_^2^ = 0.08, or for happy faces, *F*(2, 70) = 0.53, *p* = 0.591, *η*_p_^2^ = 0.02, but the main effect was significant for neutral faces (Table 5), *F*(2, 70) = 8.92, *p*_Bonf‐corrected_ < 0.003, *η*_p_^2^ = 0.20. Including age as a covariate eliminated the main effect of stimulus type for neutral faces (*p* = 0.226); however, no significant correlations were found between age and any of the stimulus types for latency values (all *p* values > 0.398). BDI‐II scores did not modify the main effect of stimulus type for latency.

**TABLE 5:** N170 latency results. Results of the post hoc (paired‐samples) *t* tests investigating the stimulus type effect for neutral condition. The direction of each significant comparison (e.g., DEV > CONT) is indicated. For N170, a shift toward negative polarity indicates a larger amplitude.

	Comparison	*t*	*p*_FDR‐corrected_	95% CI	Cohen's *d*
Neutral	DEV vs. STD	1.53	0.134	[−0.45, 3.19]	0.11
	DEV > CONT	3.59	**0.003**	[1.79, 6.45]	0.32
	STD > CONT	2.99	**0.008**	[0.88, 4.62]	0.21

*Note:* Degrees of freedom = 35 *t* values, *p* values (FDR‐corrected), and Cohen's d for effect size are reported. Significant *p* values are marked in bold.

Abbreviations: CONT = control stimulus, DEV = deviant stimulus, STD = standard stimulus.

Paired sample *t* tests found no difference in response latency between deviant and standard neutral faces (*p*_FDR‐corrected_ = 0.134, BF_10_ = 0.52), but control neutral faces had shorter latency than deviant neutral faces (*p*_FDR‐corrected_ = 0.003, BF_10_ = 31.63) and standard neutral faces (*p*_FDR‐corrected_ = 0.008, BF_10_ = 7.59).

## 4 Discussion

The present study investigated whether early brain activity associated with the perceptual processing of facial expressions—as indexed by the P1 and N170 components of ERPs—reflects deviance detection within the predictive coding framework. When physical stimulus features and deviant stimulus probability were controlled, only the N170 amplitude in response to neutral deviant faces was robustly associated with prediction error. Next, we will discuss the results in detail.

Consistent with prior research using different stimulus paradigms (Kaffes et al. 2023; Kimura et al. 2009; Kovarski et al. 2017; Li et al. 2012), we observed larger N170 amplitudes for deviant compared to both standard and control stimuli. This pattern indicates that the deviant response reflects not only stimulus rarity but also a violation of the predictive context established by the standards. Our flip‐flop design, which reversed the roles of stimuli as standards and deviants, allowed direct comparisons of physically identical stimuli presented in different predictive contexts (e.g., sad faces as standards vs. deviants). Crucially, after controlling low‐level stimulus features and stimulus probability, only neutral faces demonstrated a reliable prediction error signal as indexed by the N170 amplitude. N170 amplitude in response to happy faces did not show a clear difference between standard and deviant stimuli (Bayes factor = 1.04, indicating only very weak evidence), with a difference observed only between deviant and control stimuli. For sad faces, the N170 amplitude did not significantly differ between any of the stimulus types. This pattern suggests that, for happy faces, response amplitude is mainly influenced by stimulus context rather than stimulus probability, whereas for sad faces, neither factor appears to significantly modulate the response. Our findings further indicate that predictive deviance detection operates most efficiently for neutral faces, while for emotional faces, predictive processing seems to be disrupted. This may reflect an adaptive mechanism in which predictive coding functions optimally in emotionally neutral contexts, whereas emotional expressions may engage additional cognitive or affective processes that interfere with or override prediction‐based mechanisms.

We also analyzed the data by comparing responses to standard and deviant stimuli within each oddball block, i.e. comparing physically different stimuli (e.g., neutral standard and happy deviant, Supplementary materials). We calculated differential responses by subtracting standard from deviant responses and tested these against zero, following the analytical approach used in several previous studies (e.g., Wu et al. 2017;  Zhao and Li 2006). This analysis revealed a significant differential N170 response to happy, but not sad, deviant faces when contrasted with neutral standard faces. This finding aligns with Wu et al. (2017), who also reported greater differential responses to happy compared to sad deviant faces in healthy participants. One possible explanation for the enhanced detection of happy deviants is their more distinctive physical features—such as an open‐mouth smile revealing teeth—compared to the more subtle characteristics of sad expressions. However, Zhao and Li (2006) observed the opposite pattern, reporting larger differential response to sad than happy deviant faces. Their study, notably, used only a single facial identity, which may have facilitated change detection relative to studies like the present one that employ multiple facial identities (four in our case). Divergent findings in previous studies may, in part, stem from inadequate control of low‐level stimulus features—an issue we addressed in the present study by employing the flip‐flop oddball design.

We found no significant amplitude differences for either the P1 or N170 components when comparing responses to neutral deviant faces and emotional standard faces (Supplementary materials), similar to previous studies (Kreegipuu et al. 2013; Zeng et al. 2022). The contradictory results from the two analyses strategies likely stem from differences in what the analyses capture. When physically different stimuli are compared (e.g., happy vs. neutral faces), the observed effects may primarily reflect differences in low‐level physical features of the stimuli, which naturally influence perceptual processing and attention. In contrast, comparisons between physically similar stimuli (e.g., neutral faces as standard vs. deviant) more directly isolate predictive coding mechanisms and deviance detection by minimizing confounding effects of physical differences. Thus, analyses that do not control physical stimulus properties may conflate predictive processing with stimulus‐driven perceptual and attentional effects, leading to divergent findings. This highlights the necessity of carefully controlling stimulus features to dissociate prediction error signals from other cognitive processes involved in face perception.

Although N170 amplitude to neutral faces was modulated in a manner consistent with prediction error signaling, no such modulation was observed for N170 latency. This null finding aligns with previous studies (Li et al. 2012; Liu et al. 2016; Zhao and Li 2006), which similarly reported that deviance detection does not necessarily impact the latency of the N170 component. Interestingly, we found that control stimuli elicited the shortest latencies compared to both deviant and standard stimuli. To our knowledge, only one prior study employing an equiprobable control condition has reported N170 latency results (Li et al. 2012), and it found no significant latency differences among deviant, standard, and control responses. We speculate that the shorter latencies for control stimuli may reflect more fluent processing of continuously varying input in the control condition, in contrast to the oddball condition where repetitive standard stimuli are occasionally interrupted by deviants. This intermittent disruption may increase processing demands by engaging prediction‐related mechanisms. From a theoretical standpoint, prediction errors are thought to trigger iterative model updating within a hierarchical processing architecture (Friston 2010), a process that could, in principle, delay neural responses. However, such latency effects are not reliably observed in empirical EEG studies—and in the present study, no significant latency differences were found between standard and deviant stimuli either.

For P1, the result pattern was different from that to N170, because we found no amplitude modulation of P1, when responses to physically identical standard and deviant stimuli were compared. When responses were contrasted between neutral and emotional faces within each oddball condition, a significant differential response was observed only for P1 amplitude to sad deviant faces presented among neutral standards, although only weak evidence was found (BF_10_ = 1.05). This finding may reflect the composition of the sample, which included a majority of participants with clinical depression. Notably, in a partially overlapping sample, Ruohonen, Alhainen, and Astikainen (2020) found that depressed participants exhibited enhanced P1 amplitudes specifically in response to sad facial expressions. A negative bias toward sad content is commonly observed in cognitive neuroscience studies of depression (Disner et al. 2011), aligning with Aaron Beck's cognitive theory of depression (Beck 2008).

P1 latency was modulated by stimulus type for both sad and happy faces, but not for neutral faces, with distinct patterns emerging across facial expressions. For sad faces, P1 latency was significantly shorter when presented as deviants compared to standards. This finding contradicts the common assumption that deviant stimuli elicit delayed responses due to increased processing demands associated with violated expectations (Friston 2005; Stefanics et al. 2014). Notably, the shortest latency was observed for sad faces in the control condition, presented within an ever‐changing context. This faster response may reflect more fluent processing in the absence of a stable predictive model (Wacongne et al. 2012). No significant latency differences were found between standard and deviant stimuli for happy or neutral faces. Overall, these results suggest that P1 latency does not robustly index prediction error for any of the facial expressions examined. This is in line with previous studies showing no latency modulation for P1 to emotional faces presented either in oddball (Gayle et al. 2012; Kimura et al. 2012; Petro et al. 2023; Stefanics et al. 2012; see however, Liu et al. 2015) or other stimulus conditions (Batty and Taylor 2003; Herrmann et al. 2002; Li 2021).

In the present study, no robust effects of depression were observed on ERP components when BDI‐II scores were included as covariates in the analyses. However, depressive symptom severity attenuated the effect of stimulus type on both P1 latency and N170 amplitude. Follow‐up analyses revealed no significant correlations between BDI‐II scores and ERP measures, suggesting that the modulation by depressive symptoms may not reflect a linear relationship with symptom severity. Previous studies using group comparisons have shown alterations in processing emotional faces in depression on both P1 (Ruohonen, Alhainen, and Astikainen 2020; Zhao et al. 2015) and N170 (Xin et al. 2021; Zhao et al. 2015). As mentioned before, our data were partially the same as in a study by Ruohonen and colleagues (2020), which compared the ERPs to task‐irrelevant facial expressions with an oddball task between depressed participants and a healthy control group. They found an enlarged P1 amplitude to sad faces in depressed participants compared with the healthy group, and the P1 amplitude decreased to a normal level for depressed participants who recovered after a psychological intervention, suggesting that P1 amplitude was modulated by depressive symptoms in a state‐dependent manner.

N170 amplitude, but not that of P1 (see also Fernandes et al. 2023), associated with age. Correlation was found for each stimulus type and each facial expression, reflecting a general increase of N170 amplitude with aging. Enlarged N170 amplitude in older adults has been reported in previous literature (Fernandes et al. 2023; Gonçalves et al. 2018; Tsolaki et al. 2017). One possible explanation for this finding could be the additional neural resources required to encode the structural information in faces, potentially related to cognitive compensation at the neural level that develops across adulthood (Fernandes et al. 2023; Kisley et al. 2007). Increased neural responses in older adults have also been linked to sensory gating deficits across various sensory modalities (e.g., Bolton and Staines 2012; Ruohonen, Kattainen, et al. 2020). However, further research is needed to clarify the functional significance of enhanced amplitudes of perceptual ERP components in aging populations.

## 5 Conclusions

We investigated whether rare changes in facial expressions modulate early perceptual ERP components, potentially reflecting visual mismatch negativity (vMMN) within the predictive coding framework. Prediction error signal was robustly found for N170 amplitude modulation to neutral faces, but not for sad or happy faces, or P1 or latencies when probability and low‐level features of stimuli were controlled. Aging, but not depression, affected the N170 amplitude. As increased age was associated with enhanced amplitudes across all stimulus types, this effect is unlikely to reflect age‐related changes in predictive face perception specifically. Instead, it more likely indicates a general age‐related modulation of perceptual processing.

## 6 Limitations

The sample size was relatively small, especially for correlational analyses, and therefore these results should be interpreted cautiously. The generalizability of the results may be limited due to the sample characteristics, as the majority of participants were female and two‐thirds (24 out of 36) were clinically depressed. Although no clear effects of depression were observed, future studies should aim to replicate these findings in samples consisting of nondepressed individuals.

## Author Contributions

**Xinyang Liu:** conceptualization, formal analysis, funding acquisition, investigation, methodology, visualization, writing – original draft, writing – review and editing. **Xueqiao Li:** conceptualization, investigation, methodology, visualization, writing – review and editing. **Piia Astikainen:** conceptualization, funding acquisition, investigation, methodology, project administration, supervision, writing – review and editing.

## Ethics Statement

The experiments were conducted in accordance with the Declaration of Helsinki. Ethical approval for the study was obtained from the ethical committee of the University of Jyväskylä. Written informed consent was obtained from all the participants before their participation.

## Conflicts of Interest

The authors declare no conflicts of interest.

## Peer Review

The peer review history for this article is available at https://www.webofscience.com/api/gateway/wos/peer‐review/10.1111/ejn.70264.

## Supporting information


**Figure S1:** **Differential responses (deviant–standard) for P1.** A) Grand‐averaged difference waveforms and corresponding topographies are shown, with the electrodes used in the analysis marked. The upper panel illustrates responses from the two oddball conditions involving sad and neutral faces: The blue line represents the comparison of sad deviants versus neutral standards, and the purple line represents neutral deviants versus sad standards. The lower panel shows the oddball conditions with happy and neutral faces: the blue line represents happy deviants versus neutral standards, and the purple line represents neutral deviants versus happy standards. The waveforms represent averaged difference signals across the selected electrodes, with shaded areas indicating variability (standard error of the mean, SEM). The rectangle indicates the time window (80–120‐ms poststimulus) used to identify individual peak amplitudes for the P1 component. Amplitudes were calculated as the mean value within a 20‐ms interval centered on each individual's peak. Corresponding topographies are presented for this same time window. B) The violin plots represent the mean, standard error of the mean (SEM), and the distribution (density) of the data. The center of the white diamonds represents the mean value, and the error bars indicate the standard error of the mean. * Indicates a significant difference from zero in differential amplitude results for P1. DEV = deviant stimulus, STD = standard stimulus.
**Figure S2:** **Differential responses (deviant–standard) for N170.** A) Grand‐averaged difference waveforms and corresponding topographies are shown, with the electrodes used in the analysis marked. The upper panel illustrates responses from the two oddball conditions involving sad and neutral faces: The blue line represents the comparison of sad deviants vs. neutral standards, and the purple line represents neutral deviants versus sad standards. The lower panel shows the oddball conditions with happy and neutral faces: The blue line represents happy deviants versus neutral standards, and the purple line represents neutral deviants versus happy standards. The waveforms represent averaged difference signals across the selected electrodes, with shaded areas indicating variability (standard error of the mean, SEM). The rectangle indicates the time window (130–210‐ms poststimulus) used to identify individual peak amplitudes for the N170 component. Amplitudes were calculated as the mean value within a 20‐ms interval centered on each individual's peak. Corresponding topographies are presented for this same time window. B) The violin plots represent the mean, standard error of the mean (SEM), and the distribution (density) of the data. The center of the white diamonds represents the mean value, and the error bars indicate the standard error of the mean. * Indicates a significant difference from zero in differential amplitude results for N170. DEV = deviant stimulus, STD = standard stimulus.

## Data Availability

The data that support the findings of this study are available from the corresponding author upon reasonable request.
