# Supplementary material for: N170 Amplitude to Rare Neutral Faces in an Oddball Condition Reflects Prediction Error
Source: Eur J Neurosci. 2025 Sep 30;62(7):e70264. doi: 10.1111/ejn.70264 (PMC12484702; doi:10.1111/ejn.70264)
Supplement: Supplementary file 1 — Figure S1: Differential responses (deviant–standard) for P1. A) Grand‐averaged difference waveforms and corresponding topographies are shown, with the electrodes used in the analysis marked. The upper panel illustrates responses from the two oddball conditions involving sad and neutral faces: The blue line represents the comparison of sad deviants versus neutral standards, and the purple line represents neutral deviants versus sad standards. The lower panel shows the oddball conditions with happy and neutral faces: the blue line represents happy deviants versus neutral standards, and the purple line represents neutral deviants versus happy standards. The waveforms represent averaged difference signals across the selected electrodes, with shaded areas indicating variability (standard error of the mean, SEM). The rectangle indicates the time window (80–120‐ms poststimulus) used to identify individual peak amplitudes for the P1 component. Amplitudes were calculated as the mean value within a 20‐ms interval centered on each individual's peak. Corresponding topographies are presented for this same time window. B) The violin plots represent the mean, standard error of the mean (SEM), and the distribution (density) of the data. The center of the white diamonds represents the mean value, and the error bars indicate the standard error of the mean. * Indicates a significant difference from zero in differential amplitude results for P1. DEV = deviant stimulus, STD = standard stimulus. Figure S2: Differential responses (deviant–standard) for N170. A) Grand‐averaged difference waveforms and corresponding topographies are shown, with the electrodes used in the analysis marked. The upper panel illustrates responses from the two oddball conditions involving sad and neutral faces: The blue line represents the comparison of sad deviants vs. neutral standards, and the purple line represents neutral deviants versus sad standards. The lower panel shows the oddball conditions with happy [file EJN-62-0-s001.pdf]

N170 amplitude to rare neutral faces in an oddball condition reflects prediction error

Xinyang Liu, Xueqiao Li & Piia Astikainen

Supplementary Materials:

Analysis of differential responses within each oddball condition

To enhance comparability with previous studies that compared response amplitudes to physically different stimuli (e.g., Astikainen & Hietanen, 2009; Kovarski et al., 2022; Zhao & Li, 2006), we calculated differential responses (amplitude, deviant minus standard) for P1 and N170 separately for each oddball condition. One-sample t-tests were conducted to compare these differential responses against zero. False discovery rate procedure was used to correct the *p*-values ($p_{\text{FDR-corrected}}$). Bayes factors are reported to indicate the strength of evidence.

For P1 (Supplementary Figure 1), the t-tests showed a significant difference from zero in the oddball condition where sad deviant faces were presented among neutral standard faces, $t(35) = 3.54$, $p_{\text{FDR-corrected}} = .004$, $BF_{10} = 1.05$, 95% CI [0.18, 0.65], Cohen's d = 0.48. No significant differences were found when happy or neutral faces were presented as deviant stimuli (*ps* > .063).

For N170 (Supplementary Figure 2), a significant difference against zero was found in the condition where happy deviant faces were interspersed with neutral standard faces, $t(35) = -5.87$, $p_{\text{FDR-corrected}} < .001$, $BF_{10} = 548.86$, 95% CI [-0.76, -0.37], Cohen's d = 0.70, while other differential responses did not differ from zero (*ps* > .060).

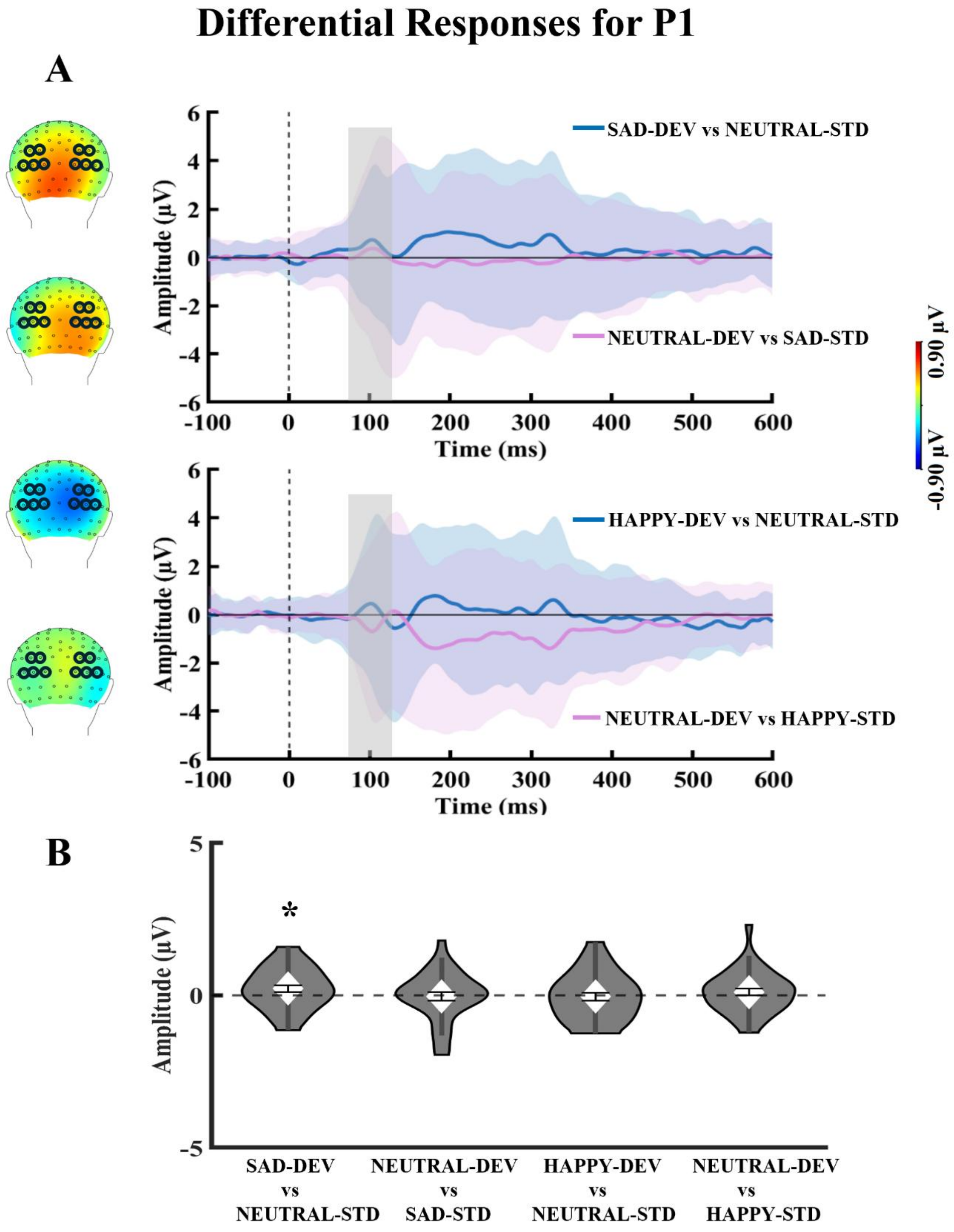

Supplementary figure 1. Differential responses (deviant – standard) for P1. A) Grand-averaged difference waveforms and corresponding topographies are shown, with the electrodes used in the analysis marked. The upper panel illustrates responses from the two oddball conditions involving sad and neutral faces: the blue line represents the comparison of sad deviants vs. neutral standards, and the purple line represents neutral deviants vs. sad standards. The lower panel shows the oddball conditions with happy and neutral faces: the blue line represents happy deviants vs. neutral standards, and the purple line represents neutral deviants vs. happy standards. The waveforms represent averaged difference signals across the selected

electrodes, with shaded areas indicating variability (standard error of the mean, SEM). The rectangle indicates the time window (80–120 ms post-stimulus) used to identify individual peak amplitudes for the P1 component. Amplitudes were calculated as the mean value within a 20-ms interval centered on each individual's peak. Corresponding topographies are presented for this same time window.

B) The violin plots represent the mean, standard error of the mean (SEM), and the distribution (density) of the data. The center of the white diamonds represents the mean value, and the error bars indicate the standard error of the mean. * Indicates a significant difference from zero in differential amplitude results for P1. DEV = deviant stimulus, STD = standard stimulus.

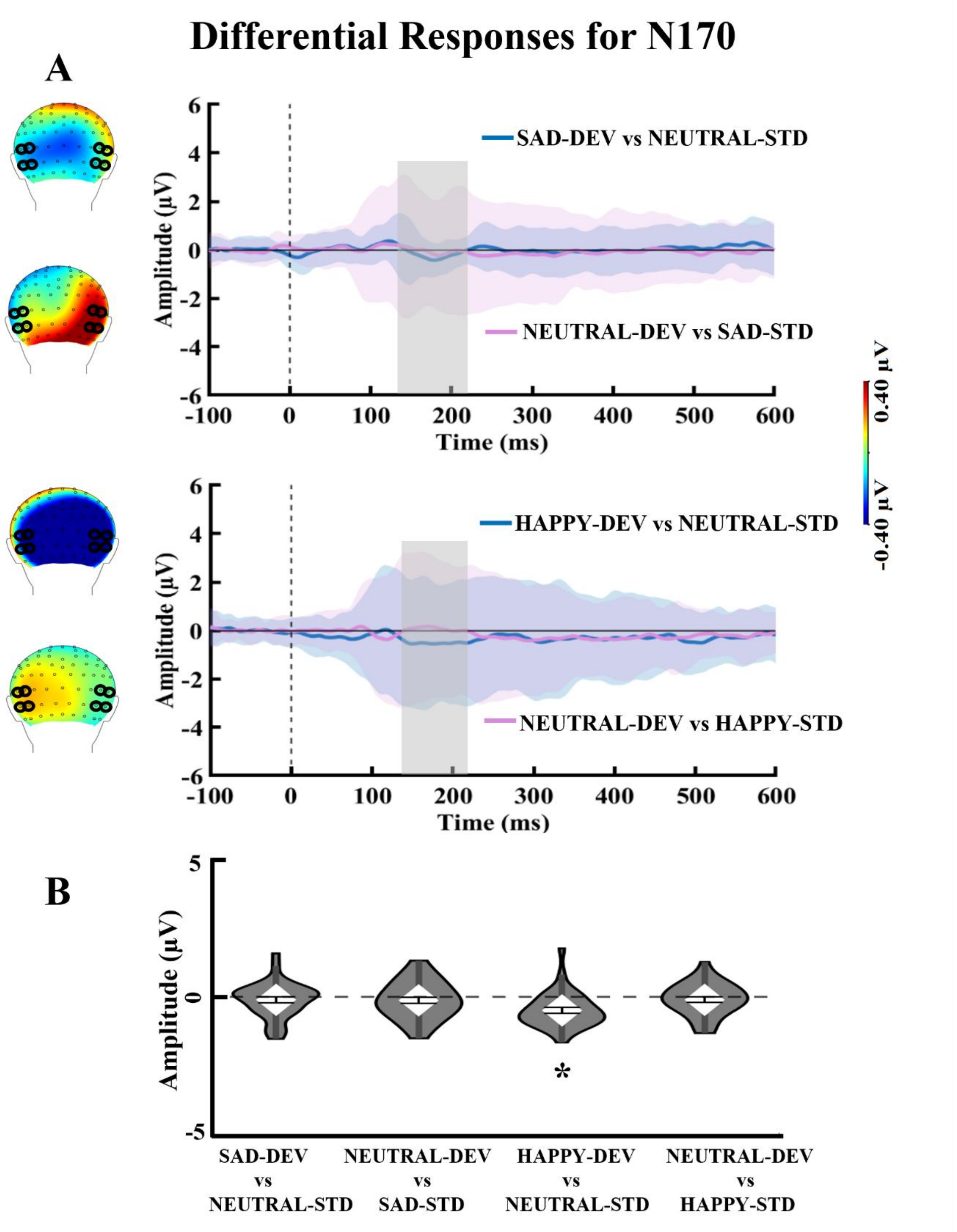

Supplementary figure 2. Differential responses (deviant – standard) for N170. A) Grand-averaged difference waveforms and corresponding topographies are shown, with the electrodes used in the analysis marked. The upper panel illustrates responses from the two oddball conditions involving sad and neutral faces: the blue line represents the comparison of sad deviants vs. neutral standards, and the purple line represents neutral deviants vs. sad standards. The lower panel shows the oddball conditions with happy and neutral faces: the blue line represents happy deviants vs. neutral standards, and the purple line represents neutral deviants

vs. happy standards. The waveforms represent averaged difference signals across the selected electrodes, with shaded areas indicating variability (standard error of the mean, SEM). The rectangle indicates the time window (130–210 ms post-stimulus) used to identify individual peak amplitudes for the N170 component. Amplitudes were calculated as the mean value within a 20-ms interval centered on each individual's peak. Corresponding topographies are presented for this same time window.

B) The violin plots represent the mean, standard error of the mean (SEM), and the distribution (density) of the data. The center of the white diamonds represents the mean value, and the error bars indicate the standard error of the mean. * Indicates a significant difference from zero in differential amplitude results for N170. DEV = deviant stimulus, STD = standard stimulus.

REFERENCE

Astikainen, P., & Hietanen, J. K. (2009). Event-related potentials to task-irrelevant changes in facial expressions. *Behavioral and Brain Functions*, *5*, 30. https://doi.org/10.1186/1744-9081-5-30

Kovarski, K., Charpentier, J., Houy-Durand, E., Batty, M., & Gomot, M. (2022). Emotional expression visual mismatch negativity in children. *Developmental Psychobiology*, *64*(7), e22326. https://doi.org/10.1002/dev.22326

Zhao, L., & Li, J. (2006). Visual mismatch negativity elicited by facial expressions under non-attentional condition. *Neuroscience Letters*, *410*(2), 126–131. https://doi.org/10.1016/j.neulet.2006.09.081
